# Effects of conservative interventions on plantar pressure in individuals with flat foot: a systematic review and meta-analysis

**DOI:** 10.1038/s41598-026-40771-5

**Published:** 2026-02-19

**Authors:** Verisheh Mahmoudiyan, Hooman Minoonejad, Fateme Khorramroo, Seyed Hamed Mousavi

**Affiliations:** Department of Sport Injuries and Biomechanics, Faculty of Sport Sciences and Health, University of Tehran, Tehran, Iran

**Keywords:** Foot pronation, Pes planus, Short foot exercise, Taping, Insole, Foot arch, Anatomy, Diseases, Health care

## Abstract

Pes planus involves the collapse of the medial longitudinal arch, altering gait and plantar pressure and leading to pain and injury. This review aimed to evaluate the effects of conservative interventions on plantar pressure in flatfoot patients. Four databases were searched from inception to 20 February 2025. Two reviewers screened studies using predefined criteria and the Downs and Black scale. Eligible designs included randomized controlled trials, quasi-experimental, and observational studies. Mean differences with 95% CIs were synthesized using a random-effects model, and evidence strength was graded using modified van Tulder criteria. Twenty-eight studies (5–80 participants; total *n* = 815) involving young to middle-aged adults with flexible flatfoot were included, and most showed moderate methodological quality. Exercise significantly reduced medial-heel pressure (MD − 12.61; 95% CI − 21.23 to − 3.98; strong evidence; 2 RCTs) but showed no significant change in midfoot pressure (MD 0.62; 95% CI − 4.41 to 5.65; 2 RCTs). Taping reduced medial-forefoot peak pressure (MD − 3.40; 95% CI − 5.78 to − 1.03; strong evidence; 5 studies) and did not change lateral-heel pressure (MD -1.26 ; 95% CI -5.11 to 2.58; strong evidence; 5 studies). Taping increased second-toe pressure by 28.2% (MD 3.69; 95% CI 1.64 to 5.75; 2 studies) and third-to-fifth-toe pressure by 34.7% (MD 3.21; 95% CI 1.85 to 4.56; 2 studies), while reducing middle forefoot by 18.2% (MD − 6.33; 95% CI − 8.95 to − 3.71; moderate evidence; 2 studies) and lateral forefoot by 12.4% (MD − 1.24; 95% CI − 2.14 to − 0.34; moderate evidence; 3 studies). Insoles increased medial-midfoot pressure by 61% (MD 29.15; 95% CI 17.23 to 41.06; 2 studies) but did not significantly change first-metatarsal (MD 8.79; 95% CI − 11.75 to 29.33; 2 studies) and lateral-midfoot pressures (MD 5.93; 95% CI − 26.31 to 38.16; 2 studies). Overall, conservative interventions may modify plantar-pressure distribution and provide short-term relief (primarily from acute/single-session taping and insole studies, with exercise effects observed over periods up to 8 weeks), but long-term efficacy remains unclear.

**Protocol registration**: CRD42024621107.

**Supplementary Information:**

The online version contains supplementary material available at 10.1038/s41598-026-40771-5.

## Introduction

Foot pronation is a natural and necessary component of normal gait, facilitating shock absorption and adaptation to uneven surfaces through heel eversion, forefoot abduction, and dorsiflexion. Coordinated subtalar and midtarsal motion absorbs ground reaction forces during weight-bearing, particularly from heel-strike to midstance^1,2^. A physiological degree of pronation facilitates efficient locomotion by ensuring balanced pressure transfer from the heel to the lateral forefoot and hallux during gait^3^. In contrast, excessive or prolonged pronation beyond midstance can disrupt lower-limb alignment and increase the mechanical load on soft-tissue and osseous structures^4^, predisposing to overuse injuries^5^.

Plantar pressure measurement quantifies this load distribution between the foot and the ground and provides insight into biomechanical deviations between healthy and pronated or flat feet. In pronated feet, medial midfoot and forefoot regions experience abnormally high pressure due to arch collapse and impaired load transmission^6^. These measurements are valuable for assessing foot health^7^, with applications in gait analysis, footwear design, sports biomechanics, and injury prevention. Clinicians use plantar pressure analysis to diagnose lower extremity disorders and evaluate gait abnormalities^8^. In individuals with flat feet, abnormal pressure distribution can lead to discomfort, which may progress to pain not only in lower limb but also lower back^9^ and lead to upper limb deformities^10,11^ if untreated, as plantar pressure serves as an early biomechanical indicator of foot dysfunction and overuse injury risk^2,12,13^.

Foot excessive pronation and flatfoot are prevalent conditions in middle-aged adults, often leading to discomfort and progressive functional limitation if unmanaged. Treatment strategies include conservative and invasive approaches^14^. With non-invasive approaches—such as orthotics, taping, exercise therapy, gait retraining^15^, and footwear modification—being preferred for mild to moderate cases^3^. These interventions aim to restore optimal foot mechanics, reduce pain, and prevent secondary musculoskeletal complications^16,17^. Modifying plantar pressure distribution is therapeutically significant as it directly addresses abnormal loading patterns that contribute to pain and functional impairment.

The standard conservative management for flatfoot combines exercise therapy and custom foot orthosis^18–20^. While insoles help redistributing pressure and correct alignment^21^, evidence on their long-term efficacy remains inconclusive^22–24^, particularly concerning pain associated with elevated plantar pressure^25,26^. Therefore, objective assessments like plantar pressure analysis should complement subjective comfort measures in evaluating intervention outcomes^27^.

Targeted strengthening of intrinsic foot muscles and the Tibialis posterior has demonstrated potential in mitigating overpronation and enhancing medial arch support^28^, whereas athletic taping provides immediate mechanical control by reinforcing the arch and redistributing pressure laterally^3,29–31^. Despite these findings, the comparative effectiveness of various conservative interventions on plantar pressure outcomes remains unclear^32^.

While numerous studies have examined conservative treatments’ effects on plantar pressure in pronated feet, no meta-analysis has simultaneously compared the relative effects of specific conservative modalities on plantar pressure distribution in individuals with flatfoot. Our study addresses this gap through a systematic review and meta-analysis evaluating how conservative interventions alter plantar pressure distribution in this population.

## Methods

The review was reported following the PRISMA 2020 statement, and methodological steps were guided by PERSiST recommendations for physiotherapy systematic reviews^33^. Additionally, the protocol was registered with PROSPERO (CRD42024621107).

### Search strategy

Relevant studies were identified through four electronic databases: PubMed (462 studies), Web of Science (1000 studies), Scopus (1013 studies), and Embase (1162 studies). The search was run to extract studies from inception to 20 February 2025. Key terms have been used in the search strategy (Table S1) and were based on broad terms and related synonyms targeting 3 categories:

#1 “pronation” OR “pronated foot” OR “pronated feet” OR “rear foot” OR “flat foot” OR “flatfoot” OR “flat feet” OR “flatfeet” OR “pes planus” OR “arch collapse” OR “planovalgus” OR “flat arched feet” OR “pes planovalgus” OR “eversion” OR “inversion” OR “low arched feet” OR “excessive calcaneal eversion” OR “medial longitudinal arch”.

#2 “Shoes” OR “footwear” OR “insole” OR “orthosis” OR “taping” OR “tape” OR “exercise” OR “training” OR “intervention” OR “therapeutic exercise” OR “physical therapy” OR “modification” OR “treatment” OR “retraining” OR “conservative”.

#3 “Pressure”.

#1 AND #2 AND #3.

Two reviewers (VM and SHM) independently screened 2094 records retrieved from the database search. Cohen’s Kappa for agreement on the relevance of records was 0.80, indicating substantial to almost perfect agreement. Search strategies of related systematic reviews published were also checked. To ensure the identification of all relevant studies, reference lists of appropriate narrative and systematic reviews two studies were added by hand-search. Studies identified through this process were included only if they met the pre-defined PICOS criteria and had not been captured by the original electronic search.

### Eligibility criteria

Each search was carried out independently according to established criteria for selecting studies and data extraction forms.

The inclusion criteria were as follows: Written-English studies that examine conservative interventions except braces or out-of-shoe orthosis on plantar pressure distribution in individuals with flat feet, measuring this variable before and after the interventions. This included both experimental and semi-experimental research published up to 20 February 2025, including studies with and without a control group. The exclusion criteria were as follows: studies involving healthy participants, case reports, and investigations on individuals without flat feet. Studies were restricted to those published in English to ensure accurate data extraction and synthesis, although this may introduce language bias by potentially excluding relevant non-English publications. In addition, gray literature (e.g., theses, dissertations, conference abstracts) was excluded due to limited methodological transparency and the absence of peer review. Therefore, only peer-reviewed published studies were included to ensure methodological consistency and reliability.

### Study selection

The study selection process was conducted in accordance with the PRISMA guidelines. Following the removal of duplicates, the titles and abstracts of all identified records were screened independently by two reviewers (VM and SHM) against the predefined eligibility criteria. This was followed by an independent full-text assessment of potentially relevant articles. Inter-rater reliability for both screening phases was quantified using Cohen’s κ statistic. A κ value of ≥ 0.80 was predefined as indicating strong agreement. Any disagreements at any stage were resolved through discussion until a consensus was reached with the assistance of an independent third reviewer (HM).

### Quality assessment

All included studies were evaluated for methodological quality using the Downs and Black Quality Index (DBQI)^34^, applying 15 relevant items for non-RCTs and the full 27-item scale for RCTs. Two reviewers (VM and SHM) independently scored each study. Any discrepancies in scoring between the two reviewers were first resolved through discussion. When consensus could not be reached through discussion alone, a third reviewer (HM) was consulted to make the final decision. Inter-rater reliability was evaluated using percentage agreement and Cohen’s kappa statistics. The results of the inter-rater agreement are reported in the Results section.

### Data collection

A standardized, piloted data extraction form was developed a priori to ensure consistent data collection. One reviewer (VM) extracted the following data from all included studies: author, year, study design, participant characteristics (e.g., sample size, sex, age, height, weight, BMI), intervention details, foot pressure measurement tools, methods of measuring the foot arch, and the primary outcome of peak and mean plantar pressure in all foot regions.

To ensure accuracy, a second reviewer (SHM) independently cross-checked all extracted data. Any discrepancies were resolved through discussion and consensus between the two reviewers, and disagreements at any stage were resolved through discussion until a consensus was reached with an independent third reviewer (HM). No quantitative transformations of data (e.g., conversion of medians to means) were required for the meta-analysis.

### Synthesis of results

Mean variations and 95% confidence intervals (CI) were computed using a random effects model in RevMan version 5.3. A meta-analysis was performed when at least 2 studies investigated the same outcome measure using comparable methods. For continuous outcomes, mean differences (MD) were estimated via the inverse variance method. The degree of statistical diversity in the combined data was measured by I^2^ statistics (> 50% as substantial heterogeneity) and corresponding P-values (*P* < 0.05). A sensitivity analysis was conducted by removing each study in turn to test the stability of the results^35^. We performed subgroup analyses by intervention type and study design. Study quality scores were not used to weight studies in the meta-analysis; they were applied solely to classify the strength of evidence (strong, moderate, and limited). Publication bias is assessed using visual inspection of funnel plots and Egger’s regression test when at least 10 studies were available for a given outcome. Results were achieved by means of levels of evidence as defined by van Tulder et al.^36^ modified by Khorramroo et al.^37^ (Table 1).

**Table 1:** Definitions of the modified van Tulder evidence framework, which integrates statistical findings (e.g., significance and consistency of pooled effects) with methodological quality ratings of the included studies to determine the overall level of evidence.

Level of evidence	Description
Strong evidence	Pooled results from three or more studies, including a minimum of two high-quality studies which are statistically homogenous (*p* > 0.05)- may be associated with a statistically significant or non-significant pooled results.
Moderate evidence	Statistically significant pooled results from multiple studies, including at least one high-quality study, which are statistically heterogeneous (*p* < 0.05); or from multiple low- or moderate-quality studies which are statistically homogenous (*p* > 0.05); or statistically insignificant pooled results from multiple studies, including at least one high-quality study, which are statistically homogenous (*p* > 0.05).
Limited evidence	Results from multiple low- or moderate-quality studies which are statistically heterogeneous (*p* < 0.05); or from one high-quality study.
Very limited evidence	Results from one low- or moderate-quality study.
Conflicting evidence	Pooled results that are insignificant and from multiple studies, regardless of quality, which are statistically heterogeneous (*p* < 0.05, i.e., inconsistent).

## Results

**Fig. 1:**
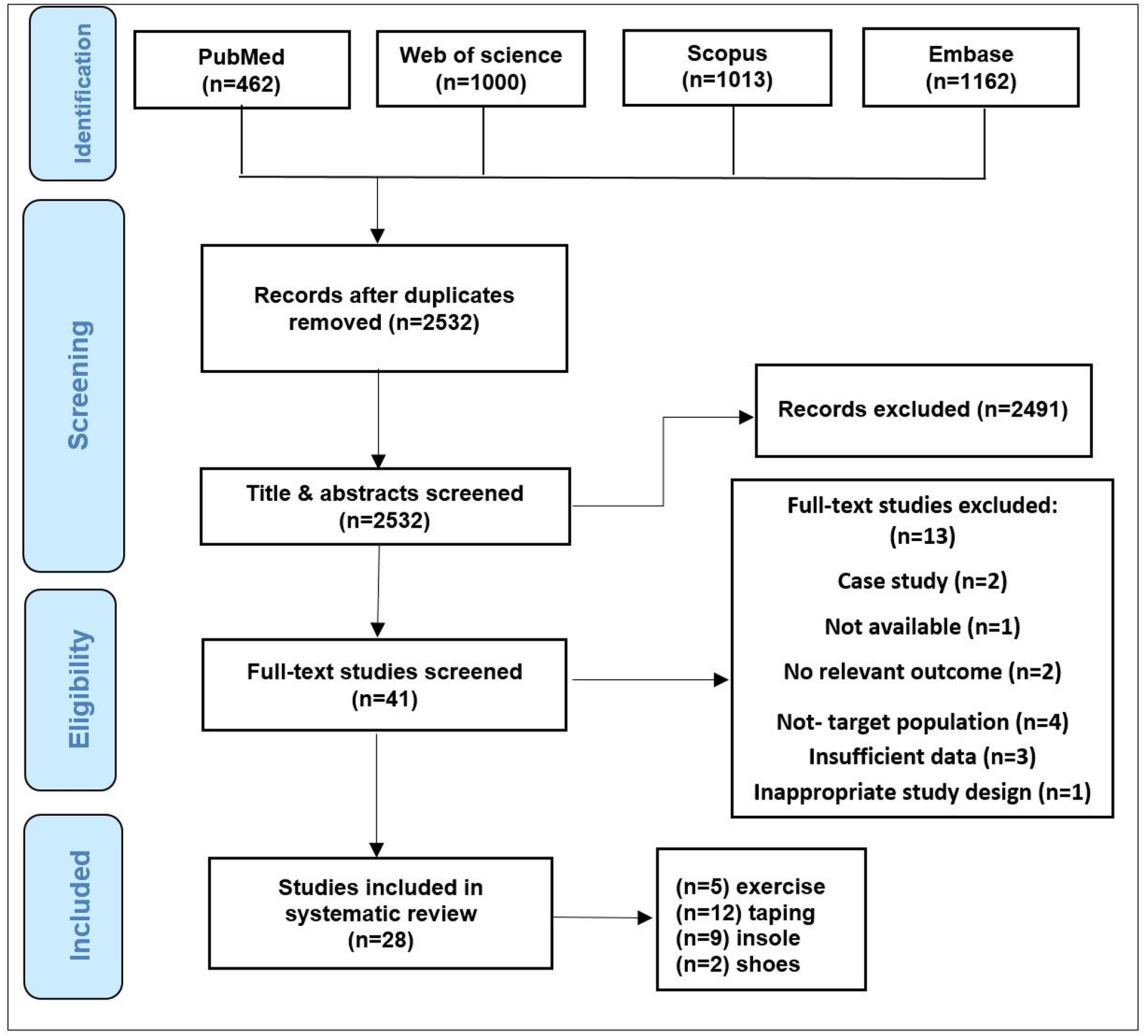
Flow chart of study selection process.

The main literature search yielded a total of 3637, from which 2532 remained after duplicate removal. A total of 1563 studies were excluded due to not meeting the inclusion criteria, and 28 were included after screening of titles and abstracts Fig. 1 shows the flow diagram, summarizing the selection process and the number of studies excluded at each stage.

### Study characteristics

Table 2 summarizes the characteristics of the included studies. There were 24 non-RCT studies^21,30,38–59^ and 4 RCTs^20,60–62^ assessing the effects of conservative treatment on plantar pressure distribution in individuals with pronated feet.

**Table 2:** Study characteristics.

Author	Study design	Subject	Sex	Age (years)	Weight (kg)	Height (cm)	BMI	FP measurement tool	Type of intervention	Method of measuring arch of the foot	Task
Da-bee Lee et al. 2016 (1)	RCT	16 young flexible pes planus	7 M+9 F	exp:24.9 ± 2.9 con:24.4 ± 7.0	exp:62.0 ± 7.4, con:61.1 ± 7.0	exp:169.5 ± 7.0 con:170.5 ± 5.9	exp:21.5 ± 1.1 con:21.0 ± 1.6	Foot scanner	Exercise	NDT	Static
Chutimon Panichawit et al. 2015 (2)		5 participants with flexible flatfoot	3 M+2 F	28.30(11.46) 18–50 yrs.	62.80(11.00)	165.60(11.60)		Force distribution platform with one camera	Exercise		Dynamic/walking
Banu Unver et al. 2019 (3)	Quasi experimental study	41 participants with pes planus	16 M + 25 F	18–25yrs				Pressure platform	Exercise	NDT-FPI	Dynamic/walking
Phoomchai Engkananuwat et al. 2023 (4)	RCT	52 healthy participants with bilateral flatfoot	26 M + 26 F	exp:28.73 ± 4.61 con:30.03 ± 4.36			exp:20.95 ± 1.06 con:21.24 ± 0.97	DIERS pedoscan plate	Exercise	NDT-AHI	Static
Walaa Elsayed et al. 2023 (5)	RCT	40 participants with sym flexible flatfoot	12 M + 28 F	exp:26.8(6.5) con:24.9(4.7)	exp:65.7(10.0), con:62.5(9.8)	exp:168(10) con:163(10)	exp:23.1(1.9) con:23.3(1.9)	Emend-x pressure measurement(dynamic)	Exercise	NDT	Static and dynamic
Belinda Lange et al. 2008 (6)		32 subjects with ND greater than 10 mm	10 M + 22 F	22.13(3.49) 18-32yrs	69.59(14.31) 48–95 kg	173.44(10.50) 153–191 cm		Emend -AT-2 platform system during gait	Taping	NDT	Dynamic/gait
Mar´ıa Bravo et al. 2015 (7)	Double-blind, 2 arm exp study	73 amateur runners with pronated foot		KT group:29.5(5.3) SKT group:27.62(7.1)	KT group:69.3(9.5), SKT group:73.20(7.9)	KT group:172.7(7.2) SKT group:176.0(5.6)		Biofeet an instrumented in-shoe insole system	Taping	FPI	Dynamic/walking
Weng-Sam Siu et al. 2019 (8)	A preliminary study	9 amateur runners with flatfoot	5 M + 4 F	21.11 ± 1.27 yrs.	63.45 ± 15.24 kg	166.2 ± 10.6 m	22.73 ± 3.17 kg/m^2^	FSR (force sensing resistor)	Taping	NDT	Dynamic/running
Damien Nolan et al. 2009 (9)	Cross-sectional	12 subjects ND > 10 mm	3 M + 9 F	25.92(12.32)				F-scan	Taping	NDT	Dynamic/gait
Mark W. Cornwall et al. 2019 (10)	Prospective cohort study	30 participants with foot pronation	10 M + 20 F	F:23(2.9) M:24(2.5)	F: 66.5(11.3), M:78.0(14.0)	F: 165.8(4.4) M:178.4(7.1)	F: 24.2(4.02) M:24.5(3.93)	The EMED-sf floor-mounted pressure platform	Taping	FPI	Dynamic/walking
Belinda Lange et al. 2004 (11)	Cross-sectional	60 participants ND > 10 mm	20 M + 40 F	21.5 ± 2.8(18–31)	67.0 ± 13.1(47–108)	168.8 ± 8.7(151–192)		The plantar platform Footscan	Taping	NDT	Dynamic/walking
Kieran O’Sullivan et al.2008 (12)	Repeated measures cross over study	20 healthy subjects ND > 10 mm	6 M + 14 F	22.1(+/-5)yrs.				F-scan a computerized insole sensor system	Taping	3D motion analysis	Dynamic/walking
One-Bin Lim et al. 2015 (13)		20 subjects with flexible flatfoot	8 M + 12 F	23.2 ± 2.1	57.3 ± 10.5	164.2 ± 7.2	21.1 ± 2.3	The Tekscan insole pressure system	Taping	NDT	Dynamic/treadmill walking
Bill Vicenzino et al. 2007 (14)	RCT	22 subjects ND>10 mm	7 M + 15 F	28.0 ± 7.4 yrs.				The EMED-SF capacitance transducer matrix platform	Taping	NDT	Dynamic/walking and jogging
Jinteak Kim et al. 2023 (15)		6 young adult	5 M + 1 F	21.00 ± 1.73 19–30 yrs.	71.33 ± 15.70	170.16 ± 5.87		GaitRite^®^ system	Taping	NDT	Dynamic/walking
Tim Newell et al.2015 (16)	Crossover study	25 subjects ND > 8 mm	13 M + 12 F	20.0 ± 1.0 yrs.	70.1 ± 10.2 kg	172.3 ± 6.6 cm			Taping	NDT	Dynamic/running
Meihua Tang et al. 2024 (17)		20 young female adultsNDT > 8 mm	20 F	23.6 ± 3.0 yrs.	54.9 ± 7.2 kg	160.2 ± 4.8 cm	21.3 ± 2.4 kg/m2	The Novel Pedar-X system	Taping	NDT	Dynamic/walking
Joseph M. Molloy et al. 2009 (18)		75 participants(40 low arched35 high arched)	54 M + 21 F	low arched group:26.5(6.5) yrs.	low arched group:74.5(12.1)	low arched group:171.0(6.6)	25.4(3.4) kg/m^2^	Pressure-sensing insoles	Shoes	AHI	Dynamic/walking
Cheng-Chieh Lin et al. 2018 (19)		35 participants with unilateral pronated foot	15 M + 20 F	26.2 ± 10.07 yrs.	60.28 ± 12.08 kg	162.34 ± 9.36 cm		The Pedar system	Shoes	NDT	Dynamic/walking
Rui Xu et al. 2019 (20)	RCT	80 patients with bilateral flatfoot	40 M + 40 F	exp:38.61(7.41) control:41.52(4.28)	exp:63.37(12.52), con:67.18(10.72)		exp:26.56(12.42) con:25.71(10.41)	Footscan^®^ system	Insole	FPI	Dynamic/walking
Lacey Nordsiden et al. 2010 (21)		20 physically active participants ND > 10	12 M + 8 F	Men: 19.7 ± 1.3 yrs. female:20.8 ± 1.5yrs	men:83.6 ± 12.3 kg, female:69.9 ± 14.2 kg	men: 181.5 ± 6.3 cm female:172.7 ± 11.2 cm		Pedar in-shoe pressure-measurement system	Insole		Dynamic/slow running
Simon Fuk-Tan Tang et al. 2015 (22)		10 persons with symptomatic flexible flatfoot		flat foot group: 24.8 ± 8.8 con group:25.1 ± 4.6	flat foot group: 62.6 ± 9.1, con group:59.7 ± 9.4	flat foot group:163.9 ± 6.5 con group:165.4 ± 8.2		Pedar Expert software	Insole	AHI	Dynamic/gait
Yangzheng Jiang et al. 2021 (23)		10 subjects with flexible flatfeet	8 M + 2 F		67.4 ± 10.9	167.9 ± 5.5		Xsensor FOOT and GAIT software	Insole		Dynamic/walking
Yao-Te Wang et al. 2020 (24)		26 subjects	25 M + 1 F	22 ± 1 yrs.	68.3 ± 5.4 kg	173.6 ± 2.5	22.6 ± 1.2		Insole		Dynamic/walking
Yu-ping Huang et al. 2020 (25)		15 female college students with flatfoot	15 F	19.7 ± 4.3	56.5 ± 6.7	160.9 ± 6.0 cm		F-Scan sensor	Insole		Dynamic/walking
JunNa Zhai et al. 2017 (26)		30 participants(15 with flatfoot 15 normal feet)	30 F					RSscan force plate	Insole		Dynamic/walking
Aminian (27)	Quasi - experimental	12 subjects with flatfoot	12 M	22.25 ± 1.54	72.9 ± 6.05 kg	178 ± 3.95 cm		Pedar_x system	Insole		Dynamic: walking
Banafsheh Khodaei (28)		19 flatfeet adults	17 F + 2 M	22.89 (3.49)	64.37 (8.13)	1.65 (0.051)	23.35 (1.89)	Pedar-X system	Insole	FPI	Static/dynamic: walking

The included studies demonstrated considerable heterogeneity in intervention type, duration, sample characteristics, and outcomes. Exercise-based interventions ranged from intrinsic foot muscle strengthening to comprehensive foot core programs. Taping and insole interventions were mostly short-term or cross-sectional, focusing on immediate or short-duration effects. Sample sizes varied widely, from as few as 5 to as many as 80 participants, predominantly young adults aged between 18 and 41 years, with both male and female participants included. However, the included participants all had flexible flatfoot. Overall, most interventions—particularly exercise, taping, and insoles—resulted in improved plantar pressure distribution and reduced midfoot loading, with exercise programs producing more sustained improvements in medial arch height. However, due to methodological diversity and variations in participant characteristics, the results across studies should be interpreted with caution.

### Quality assessment

Two reviewers independently assessed study quality using the DBQI tool (VM and SHM). Inter-rater reliability was quantified using Cohen’s kappa (κ = 0.82), indicating substantial agreement. The percentage agreement between reviewers was 90%. Mean DBQI scores (± SD) by study type were as follows: RCTs (24 ± 2.1), non-RCTs (11.67 ± 1.4). All discrepancies were resolved through discussion with a third reviewer (HM). Only fifteen out of 27 questions of DBQI were answered for non-RCTs based on the modified DBQI. The methodological quality of non-randomized studies was moderate to high (9–14 out of 15).The high proportion of non-randomized studies (24/28) necessitates cautious interpretation of pooled estimates due to potential residual confounding.

Table 3 Shows the results of quality assessment. The average score of eligible studies was 11.66 for cross-sectional studies^21,30,38–59^ and 24 for the RCTs^20,60–62^.

**Table 3:** Quality assessment.

Questions	Aim clearly described?	Main outcomes described in introduction or method?	Patient’s characteristics clearly described?	Interventions clearly described?	Principal confounders clearly described	Main findings clearly described?	Estimates of random variability provided for main outcomes?	All adverse events reported?	Characteristics of patients lost to follow up described?	*P*-value report for main outcome?	Subjects asked to participate representative of source population?	Subjects prepared to participate representative of source population?	Location and delivery of treatment was representative of source population?	Study participants blinded to treatment?
Study/question number	1	2	3	4	5	6	7	8	9	10	11	12	13	14
Da-bee Lee et al. 2016	1	1	1	1	2	1	1	1	1	1	1	1	1	0
Chutimon Panichawit et al. 2015	1	1	1		0	1	1			1	0	1		
Banu Unver et al. 2019	1	1	0		0	1	1			1	0	1		
Phoomchai Engkananuwat et al. 2023	1	1	1	1	1	1	1	1	1	1	1	1	1	1
Walaa ElsayEd et al. 2023	1	1	1	1	1	1	1	1	0	1	1	1	1	0
Belinda Lange et al. 2008	1	1	1		1	1	1			0	0	1		
Mar´ıa Bravo et al. 2015	1	1	1		1	1	1			0	0	1		
Weng-Sam Siu et al. 2019	1	1	1		0	1	1			1	0	1		
Damien Nolan et al. 2009	1	1	0		1	1	1			1	0	1		
Mark W. Cornwall et al. 2019	1	1	1		0	1	1			1	0	1		
Belinda Lange et al. 2004	1	1	1		1	1	1			1	0	1		
Kieran O’Sullivan et al.2008	1	1	1		1	1	1			1	1	1		
One-Bin Lim et al. 2015	1	1	1		1	1	1			1	0	1		
Bill Vicenzino et al. 2007	1	1	0		1	1	1			1	0	1		
Jinteak Kim et al. 2023	1	1	1		0	1	1			1	0	1		
Tim Newell et al.2015	1	1	1		2	1	1			1	1	0		
Meihua Tang et al. 2024	1	1	1		1	1	1			1	0	1		
Joseph M. Molloy et al. 2009	1	1	1		0	1	1			1	1	1		
Cheng-Chieh Lin et al. 2018	1	1	1		0	1	1			0	0	1		
Rui Xu et al. 2019	1	1	1	1	1	1	1	1	1	1	1	1	1	1
Lacey Nordsiden et al. 2010	1	1	1		0	1	1			1	0	1		
Simon Fuk-Tan Tang et al. 2015	1	1	1		0	1	1			1	0	1		
Yangzheng Jiang et al. 2021	1	1	1		0	1	1			1	0	1		
Yao-Te Wang et al. 2020	1	1	1		1	0	0			1	0	1		
Yu-ping Huang et al. 2020	1	1	1		0	1	1			1	0	1		
JunNa Zhai et al. 2017	1	1	1		1	0	1			1	0	1		
Aminian	1	1	1		0	1	1			1	0	0		
Banafsheh Khodaei	1	1	1		2	1	1			1	0	0		
Percentage agreement reliability	100%	100%	92%		96%	100%	96%			100%	96%	92%		

Questions	Blinded outcome assessment	Any data dredging clearly described?	Analysis adjusts for differing follow-up length?	Appropriate statistical test performed?	Compliance with interventions was reliable?	Outcome measures were reliable and valid?	All participants recruited from the source population?	All participants recruited over the same period of time?	Participants randomized treatment?	Allocation of treatment concealed from investigators and participants?	Adequate adjustment for confounding?	Losses to follow up taken into account?	Sufficient power to detect treatment effect at significance level of 0.05?	
Study/question number	15	16	17	18	19	20	21	22	23	24	25	26	27	Total
Da-bee Lee et al. 2016	0	1	1	1	1	1	1	0	1	0	1	1	0	23
Chutimon Panichawit et al. 2015		1		1		1	0	0			0			10
Banu Unver et al. 2019		1		1		1	1	0			0			10
Phoomchai Engkananuwat et al. 2023	1	1	1	1	1	1	1	1	1	1	1	1	1	27
Walaa ElsayEd et al. 2023	1	1	1	1	1	1	1	0	1	0	1	0	1	22
Belinda Lange et al. 2008		1		1		1	1	1			1			13
Mar´ıa Bravo et al. 2015		1		1		1	0	0			1			11
Weng-Sam Siu et al. 2019		1		1		1	1	0			0			11
Damien Nolan et al. 2009		1		1		1	0	1			1			12
Mark W. Cornwall et al. 2019		1		1		1	1	1			0			12
Belinda Lange et al. 2004		1		1		1	0	1			1			13
Kieran O’Sullivan et al.2008		1		1		1	1	0			1			14
One-Bin Lim et al. 2015		1		1		1	0	1			1			13
Bill Vicenzino et al. 2007		1		1		1	0	1			1			12
Jinteak Kim et al. 2023		1		1		1	0	1			0			11
Tim Newell et al.2015		1		1		1		1			1			14
Meihua Tang et al. 2024		1		1		1	1	0			1			13
Joseph M. Molloy et al. 2009		1		1		1	1	1			0			13
Cheng-Chieh Lin et al. 2018		1		1		1	0	0			0			9
Rui Xu et al. 2019	0	1	1	1	1	1	1	1	1	0	1	1	0	24
Lacey Nordsiden et al. 2010		1		1		1	0	1			0			11
Simon Fuk-Tan Tang et al. 2015		1		1		1	0	1			0			11
Yangzheng Jiang et al. 2021		1		1		1	0	0			0			10
Yao-Te Wang et al. 2020		1		1		0	1	1			1			11
Yu-ping Huang et al. 2020		1		1		1	1	0			0			11
JunNa Zhai et al. 2017		1		1		0	0	0			1			10
Aminian		1		1		1	1	1			1			12
Banafsheh Khodaei	0	1		1		1	1	1			1			14
Percentage agreement reliability		95%		100%		92%	96%	100%			100%			

Key: 1 = Yes; 0 = No. *2 = Yes; 1 = Partially; 0 = No; *= the question discussed with the third reviewer.

### Outcomes

A negative direction of effect (decrease in plantar pressure) indicates improvement, reflecting reduced loading in medial regions commonly overloaded in flatfoot. A positive direction (increase in pressure) may indicate either worsening in these regions or a redistribution of load toward the lateral foot and toes.

Publication bias could not be formally assessed due to the small number of included studies for each subgroup. Subgroup analyses were performed according to intervention type and study design, which were the only categories supported by the available data.

### Effects of exercise

**Fig. 2:**
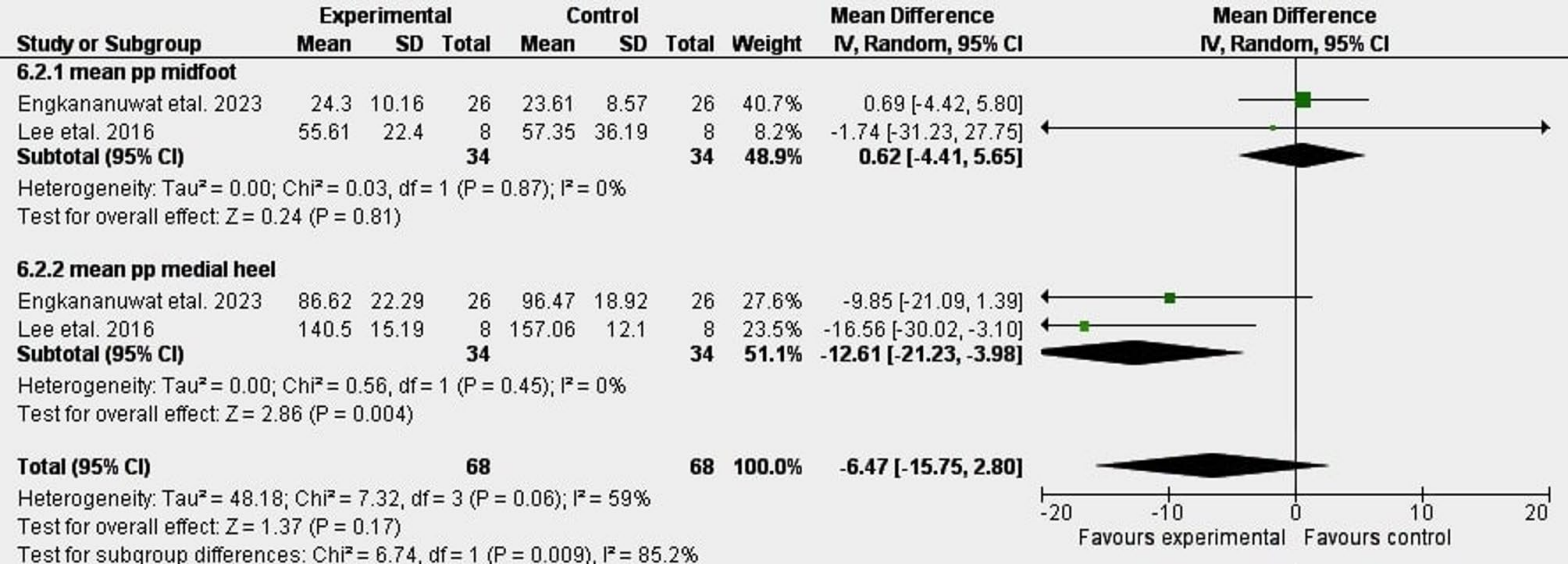
A meta-analysis of the effect of exercise.

**Fig. 3:**
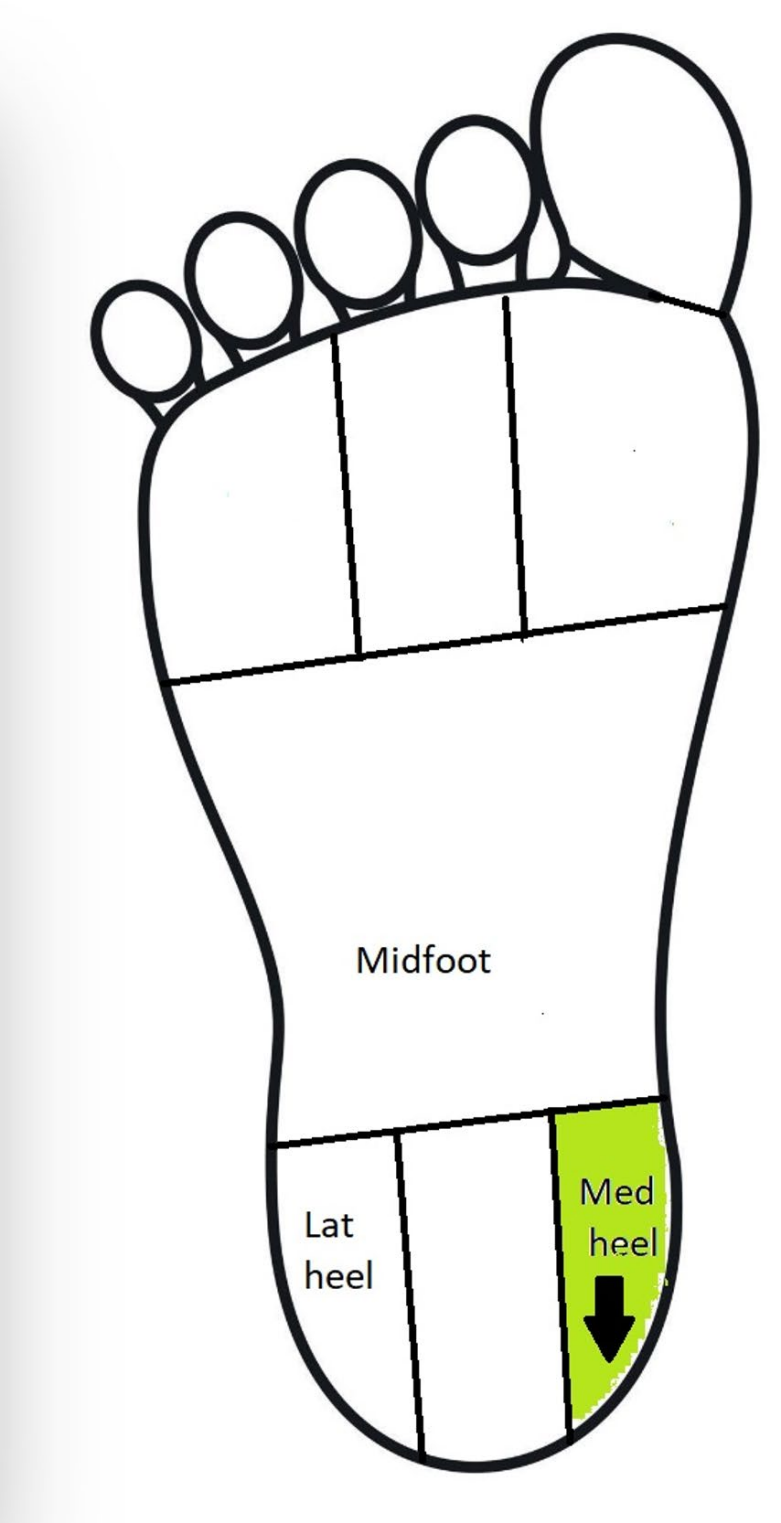
Graphical abstract of meta-analysis (mean PP).

Figure 2 shows the results of the meta-analysis showed moderate evidence of a 10% significant decrease in mean plantar pressure in medial heel MD: -12.61 [CI: -21.23, -3.98]^60,61^. Moderate evidence showed a non-significant change in mean plantar pressure in midfoot in the exercise group compared to the control group 0.62[-4.41, 5.65]^60,61^. The moderate heterogeneity suggests variation in exercise protocols and participant characteristics. Limited evidence showed increased pressure area (Figure 3)^20^.

### Effects of taping

**Fig. 4:**
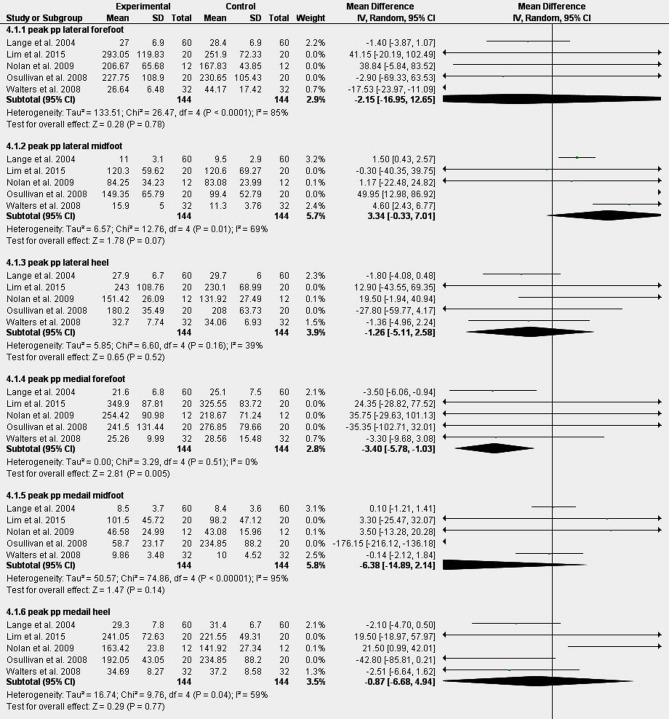

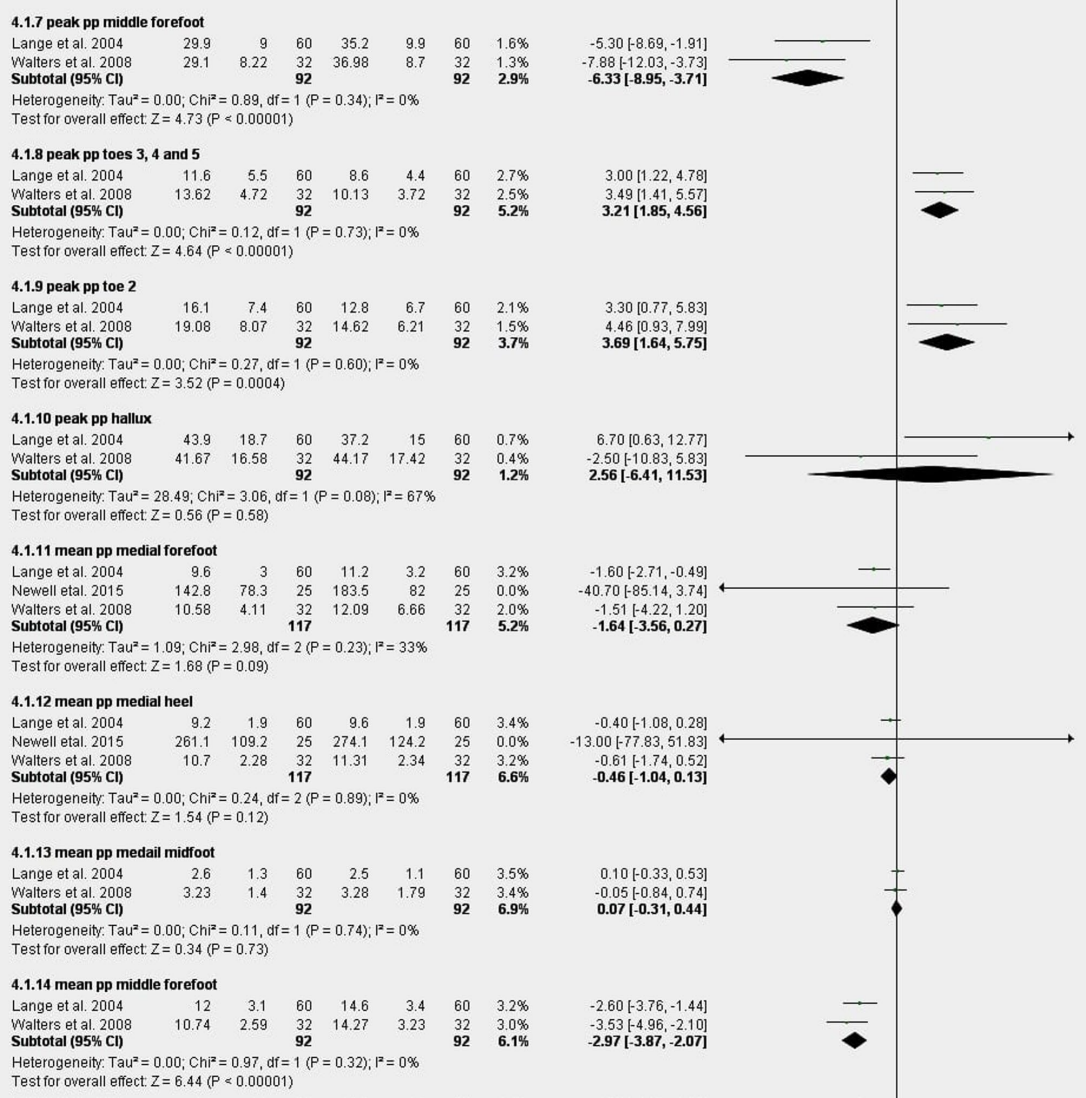

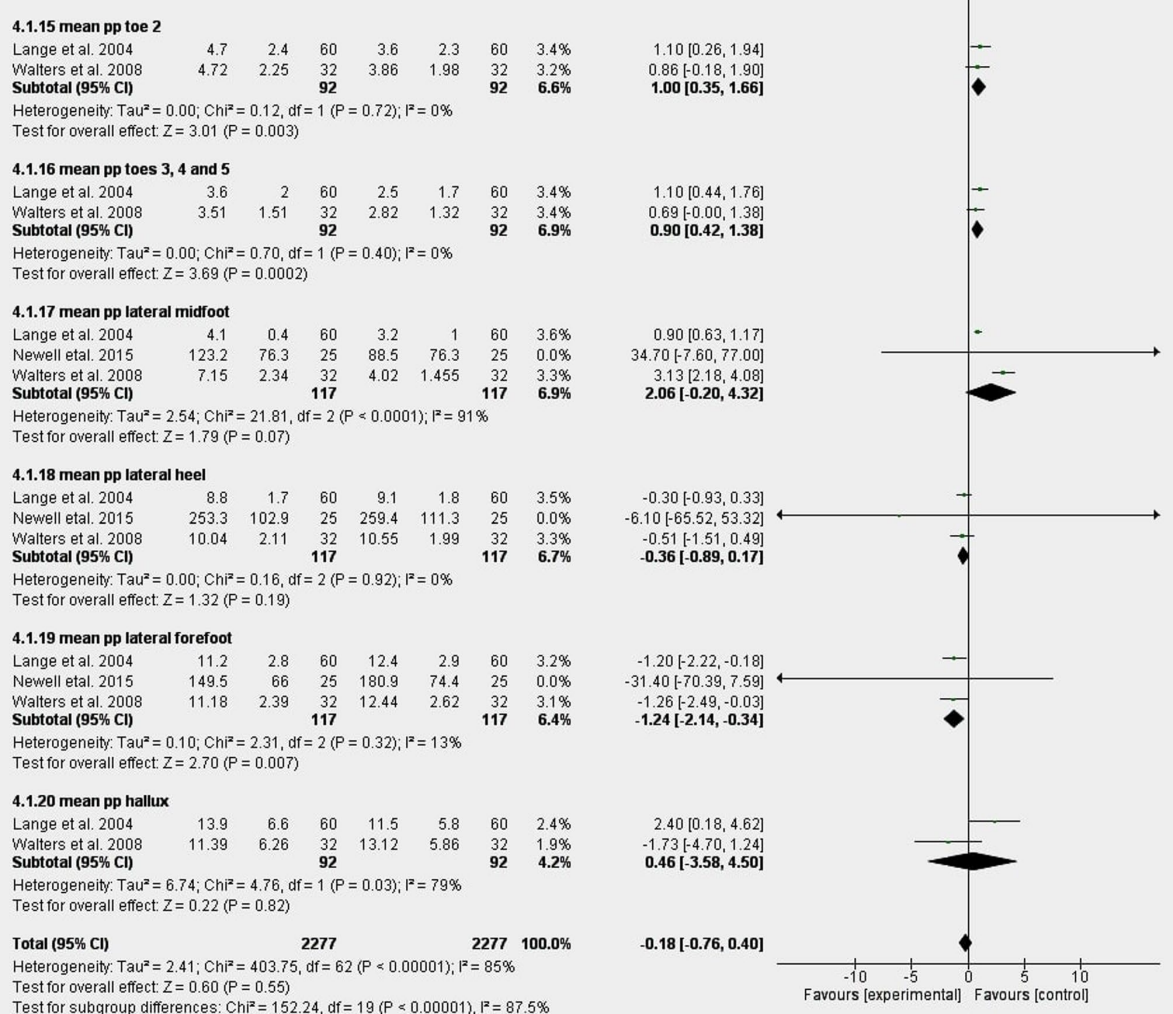
(**A**–**C**) Meta-analysis of the effect of taping for all reported foot regions.

Figure 4 Shows the results of the meta-analysis of the effect of taping for all reported foot regions. Meta-analysis showed strong evidence of a significant decrease in peak plantar pressure in medial forefoot, MD: -3.40 [-5.78, -1.03]^39,42,44–46^. There was strong evidence showing non-significant change in peak plantar pressure in the lateral heel in the taping group compared to the control group − 1.26[-5.11,2.58]^39,42,44–46^. Moderate evidence showed significant increases in peak plantar pressure of 28.2% (MD: 3.69 [1.64, 5.75]) in toe 2 and 34.7% (MD: 3.21 [1.85, 4.56]) in toes 3–5, alongside mean plantar pressure rises of 26.5% (MD: 1.00 [0.35, 1.66]) in toe 2 and 34.3% (MD: 0.90 [0.42, 1.38]) in toes 3–5. Additionally, moderate evidence showed significant reductions in plantar pressure: 18.2% (MD: -6.33 [-8.95, -3.71]) in peak middle forefoot, 21.3% (MD: -2.97 [-3.87, -2.07]) in mean middle forefoot, and 12.4% (MD: -1.24 [-2.14, -0.34]) in mean lateral forefoot^39,44,49^. Sensitivity analysis by removing the study by Walters et al.^39^ revealed that the lateral forefoot results became homogeneous instead of heterogeneous and non-significant (Fig.S1). This change may be due to the different measurement devices.

Moderate evidence indicated no significant changes in peak plantar pressure across regions, including the lateral forefoot (MD = − 2.15 [− 16.95, 12.65]), lateral midfoot (3.34 [− 0.33, 7.01]), medial midfoot (− 6.38 [− 14.89, 2.14]), medial heel (− 0.87 [− 6.68, 4.94]), and hallux (2.56 [− 6.41, 11.53]). Similarly, mean plantar pressure showed non-significant differences in the medial forefoot (− 1.64 [− 3.56, 0.27]), medial heel (− 0.46 [− 1.04, 0.13]), medial midfoot (0.07 [− 0.31, 0.44]), and lateral heel (− 0.36 [− 0.89, 0.17])^39,42,44–46,49^.

Removing Lange et al.^44^ and Walters et al.^39^ revealed that the lateral midfoot results became homogeneous, while excluding O’Sullivan et al.^45^ made them homogeneous and significant. Sensitivity analysis by removing Lange et al.^44^ or Walters et al.^39^ revealed that the medial forefoot results changed from homogeneous and significant to homogeneous and non-significant. Removing O’Sullivan et al.^45^ revealed that the medial midfoot results became homogeneous, and removing O’Sullivan et al.^45^ or Nolan et al.^42^ revealed that the medial heel results became homogeneous (Fig.S1). All changes are probably due to measurement devices, except for Nolan et al., which also has a small sample size. Evidence showed conflicting results for mean plantar pressure with taping versus control: +48.4% (MD: 2.06, 95% CI [-0.20, 4.32]) in the lateral midfoot and + 3.9% (MD: 0.46, 95% CI [-3.58, 4.50]) in the hallux^39,44,49^.

**Fig. 5:**
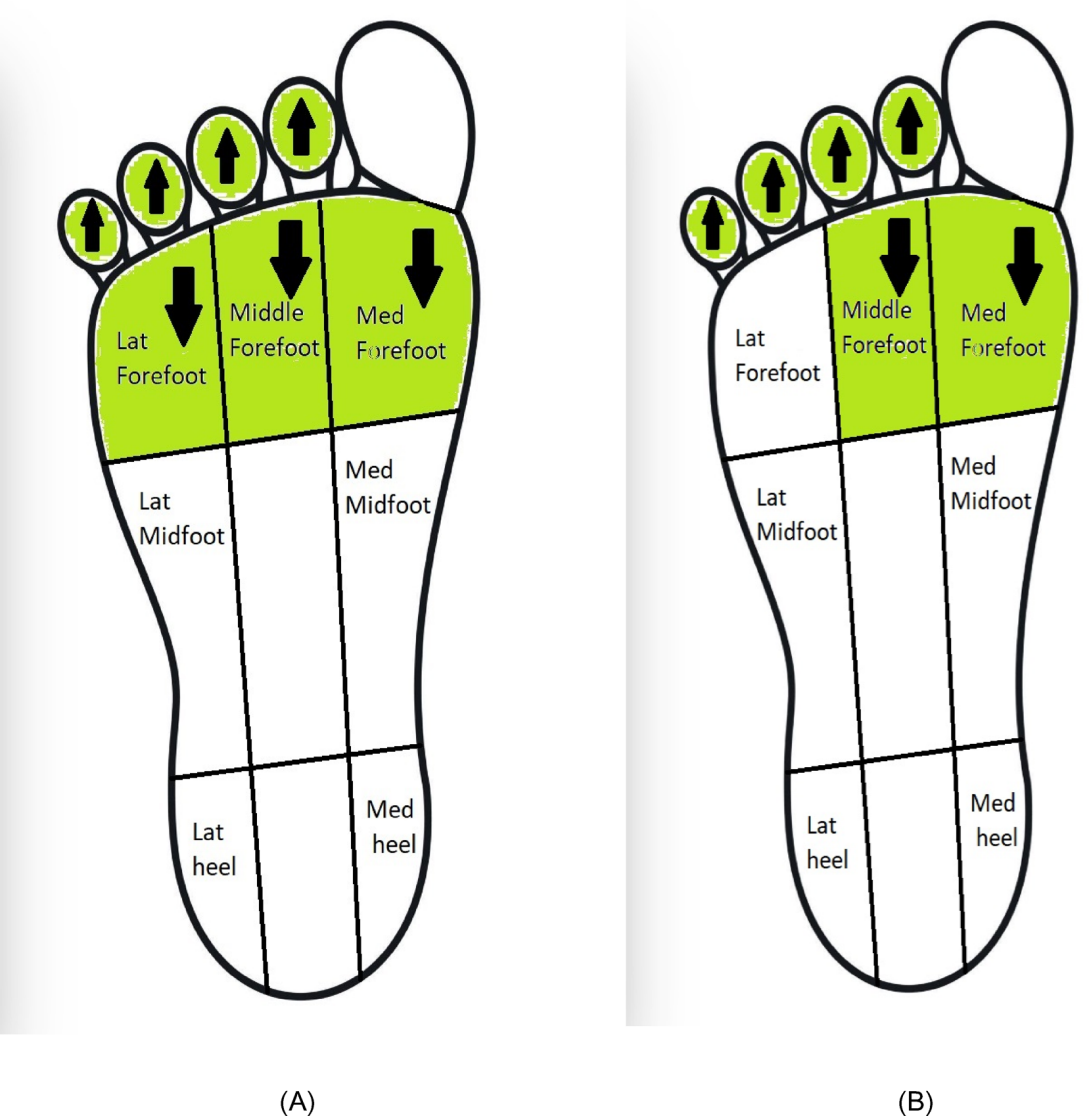
(**A**) Graphical abstract of meta-analysis (mean PP) for taping. (**B**) Graphical abstract meta-analysis (peak PP) for taping.

Limited evidence showed a decrease in pressure time in heel strike rearfoot and heel strike midfoot, and an increase in toe-off forefoot pressure time integral and toe-off midfoot pressure time integral^40^. Limited evidence showed a decrease in the integrated pressure over time and peak pressure on the medial side of the midfoot for both feet compared to the barefoot state^48^. Also, limited evidence showed increased plantar pressures in the lateral midfoot during walking and jogging. Limited evidence showed Taping could improve plantar pressure distribution, prospectively changing peak pressure of the second to fifth toe area and medial midfoot, integrated contact area, and force-time integration medial midfoot during walking (Fig. 5)^50^.

### Effects of footwear interventions

Limited evidence showed an increase in peak plantar pressure in lateral midfoot, medial forefoot, and medial midfoot in unadjusted motion control shoes compared to own shoes. Also, limited evidence showed an increase in peak plantar pressure in lateral midfoot and medial forefoot in adjusted motion control shoes compared to their own shoes^27^. Cushioning and motion control running shoes tend to increase midfoot mean plantar contact area while decreasing mean plantar pressure across the low arched foot^51^.

### Effects of insoles

**Fig. 6:**
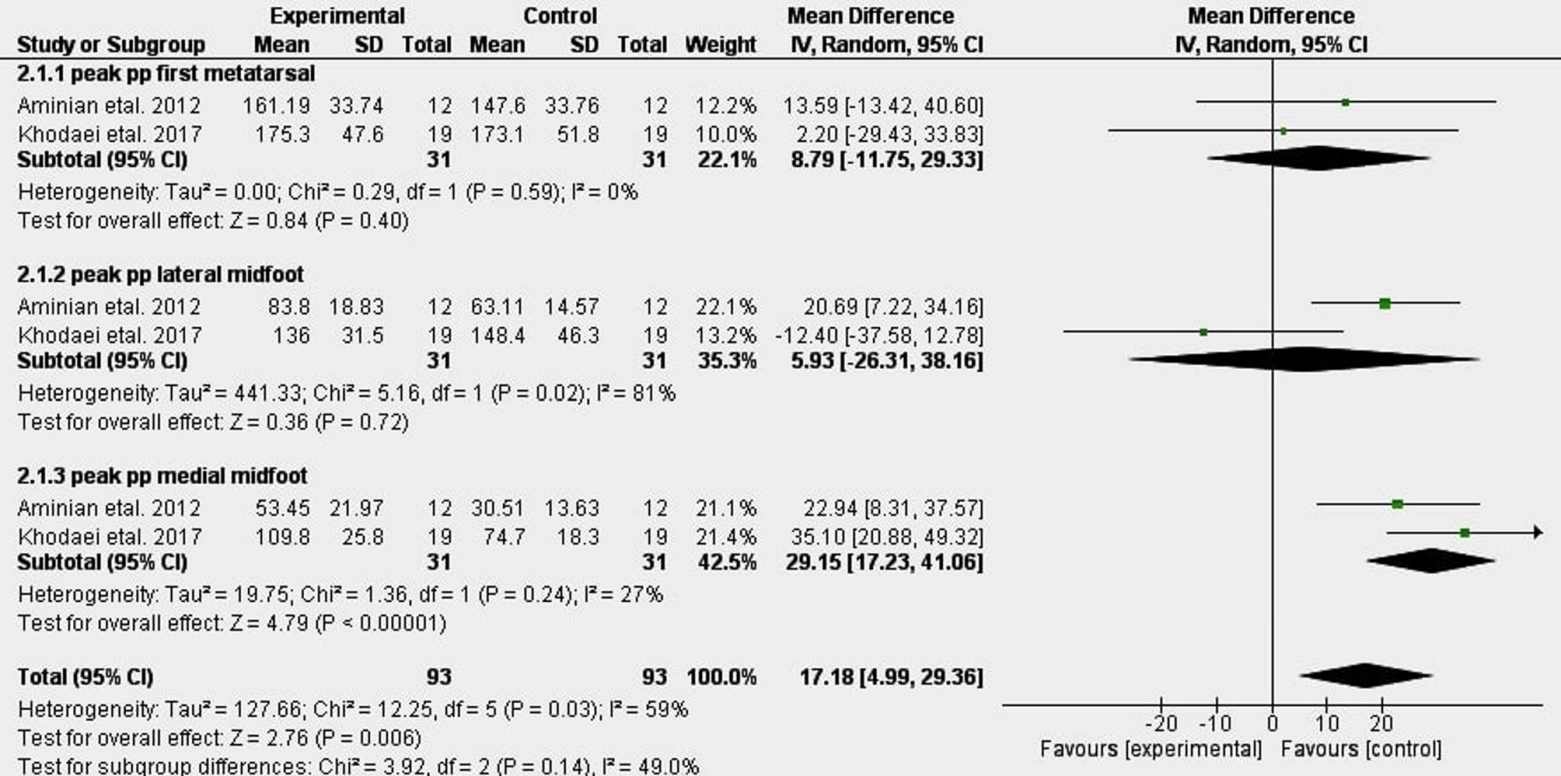
Meta-analysis of the effects of insoles.

Figure 6 shows the results of the meta-analysis for the effect of insoles. Moderate evidence showed a 61% significant increase in peak plantar pressure in the medial midfoot (MD: 29.15 [17.23, 41.06]) but a non-significant change in peak plantar pressure in the first metatarsal (MD: 8.79 [-11.75, 29.33]) and the lateral midfoot (MD: 5.93 [-26.31, 38.16]) in the insole group compared to the control group^58,59^.

Limited evidence revealed that plantar pressure redistribution insoles improve the plantar pressure distribution and gait efficiency of adults with flexible flatfeet and can be applied in clinical settings^21^. The experimental results showed that the use of textured insoles designed with different heights could not effectively improve the plantar pressure distribution and body stability in subjects with flat feet. Conversely, limited evidence showed that the use of an artificial arch effectively improved the excessive peak in pressure and poor body stability, and alleviated the problem of plantar collapse for patients with flat feet, especially in the inner part of their hallux and forefoot^55^. The peak pressure of the big toe in the arch-support insole was significantly greater than in the flat insole on the uphill and level surfaces. Limited evidence showed that the peak pressure of the metatarsals 2–4 and the contact area of the midfoot in the arch-support insole were significantly greater than in the flat insole on all surfaces^56^. Limited evidence showed Orthotic insoles could effectively improve the plantar pressure of flatfoot under different walking conditions^57^.

Conservative treatments generally redistribute plantar pressure by reducing medial arch load and increasing lateral forefoot support. Among them, exercise-based interventions (particularly foot-core strengthening) had the best results, showing the most consistent and clinically meaningful reduction in medial heel and midfoot pressure without undesirable compensatory loading elsewhere. Taping produced large but highly variable and short-term effects, while insoles showed the least favorable profile with frequent increases in medial midfoot pressure. Long-term, high-quality RCTs are needed to confirm these findings.

## Discussion

This systematic review examined conservative treatments for plantar pressure distribution in pronated feet, including exercise (5 studies, *n* = 154)^30,38,60,61,63^, taping (12 studies, *n* = 329)^39–43,45–50,64^, insoles (9 studies, *n* = 222)^21,53–59,62^, and footwear (2 studies, *n* = 110)^52,65^.

The meta-analysis indicates that taping significantly reduced peak and mean plantar pressure in medial/middle forefoot regions while increasing PP in toes 2–5, whereas exercise interventions decreased mean PP in the medial heel. Additional outcomes included dynamic/static PP, pressure-time integrals, and running-related PP measurements, providing comprehensive biomechanical insights. These preliminary results support conservative treatments for modulating pathological pressure patterns in pronated feet, which is clinically significant given their association with foot pain and dysfunction.

Despite promising results, variability exists - particularly for insoles’ region-specific effects and limited footwear data. While taping and exercise demonstrate clinical value, most benefits appear acute or short-term, small samples and short follow-ups highlight the need for larger, longer-term studies to optimize treatment protocols^39,52,63,66^. Our results, when integrated with the wider literature, strongly suggest that although conservative interventions are effective in the short term, sustained benefits most likely require ongoing application, periodic re-application, or integration into multimodal treatment programs.

### Effects of exercise

The meta-analysis demonstrated significant reductions in mean plantar pressure at the medial heel^60,61^. Because healthy gait typically exhibits a posterior-to-anterior and lateral-to-medial pressure progression, whereas pronated feet display persistent medial overloading, this medial heel offloading represents a shift toward a more physiological pattern^3^. This medial heel offloading may decrease tensile stress on the plantar fascia origin and improve rearfoot stability in pronated feet^38,63,67^. These changes align with previous reports of improved rearfoot control following short-foot and intrinsic-foot muscle training,^30,38,61,63^, though responsiveness may depend on adherence, foot morphology, and neuromuscular adaptation, and morphological changes in intrinsic muscles often require training durations longer than 8 weeks^68^. Such redistribution may also enhance dynamic stability by reducing excessive medial collapse during stance. However, a growing body of evidence reveals conflicting long-term outcomes; several high-quality studies have shown that the positive biomechanical and clinical effects gained from short-foot exercises are largely lost within 4–12 weeks after training cessation^69–71^ These divergent findings reinforce that exercise-induced improvements are highly transient and underscore the necessity of maintenance programs to preserve benefits over time. Although midfoot pressure changes were not significant overall, specific protocols (e.g., combined hip–foot exercises) achieved superior redistribution^61^, underscoring the importance of exercise selection.

### Effects of taping

Taping produced the most consistent and clinically meaningful redistribution, with a significant reduction in peak pressure in the medial forefoot (likely reducing plantar fascia and intrinsic foot structure strain)^39,42,45,46,64^, and concomitant increases in toes 2–5 and lateral regions. By shifting load laterally and distally, taping may temporarily counteract the pathological medial overload characteristic of flatfoot and improve functional alignment during gait. This medial-to-lateral and forefoot-to-toes shift represents a key therapeutic principle of taping in pronated feet. Our findings are highly consistent with multiple prior systematic reviews and meta-analyses on low-Dye and Kinesio taping techniques^72,73^, which uniformly report immediate and statistically significant pressure redistribution that is, however, transient and typically disappears within 1–6 h after tape removal. Readers should note that taping’s efficacy is short-lived, and benefits diminish post-application rapidly, cautioning against overestimating long-term effects. These acute effects are consistent with prior meta-analyses on low-Dye and Kinesio taping that similarly report immediate but transient pressure redistribution^39,43,45^ with most benefits diminishing within hours after application. Contrary to traditional assumptions, low-Dye taping did not produce global anti-pronation correction but induced localized midfoot effects^46,74^. Clinically, these short-term improvements may help reduce symptom severity during activity but should not be considered a sustained corrective intervention. The predominance of acute studies remains a major limitation for clinical translation.

### Effects of footwear interventions

Motion-control footwear increased peak pressure in the lateral midfoot and medial forefoot compared to neutral shoes^52,65^, potentially creating new high-pressure zones despite intended pronation control. Interestingly, participants perceived greater comfort and stability^52^, highlighting a disconnect between objective pressure data and subjective outcomes that warrants further investigation using dynamic pressure-gradient analysis. Such redistribution may modify lower-limb kinematics by constraining rearfoot motion, but increased lateral loading could elevate the risk of secondary discomfort or compensatory gait strategies^75^. Future discussion should consider how this pressure redistribution trade-off may influence comfort, gait efficiency, and risk of secondary pain sites, particularly during prolonged wear.

### Effects of insoles

The meta-analysis highlights variable efficacy among insole designs for managing plantar pressure in pronated feet. While moderate evidence shows no significant pressure changes at the first metatarsal and lateral midfoot^58,59^, certain designs—particularly those with artificial arches—demonstrate clinically meaningful reductions in forefoot and hallux pressures^55^. By reducing excessive medial loading, these insoles may help restore a more physiological gait pattern and alleviate strain on structures commonly overloaded in flatfoot. Textured insoles, however, appear ineffective for pressure redistribution or stability improvement^55^. Customized and prefabricated insoles showed comparable heel effects^62^. It is important to distinguish between customized and prefabricated insoles, as clinical outcomes often vary according to the degree of individualization, though some studies report comparable heel effects^62^. Some insoles mirrored the medial-to-lateral redistribution seen with taping, reinforcing this as a shared mechanism among effective conservative interventions.

Sensitivity analyses showed that pooled effects in the lateral forefoot, lateral midfoot, and medial forefoot were not robust. In each region, significance depended heavily on a small number of older, high-weight studies—particularly Walters et al.^39^ and, in some cases, Lange et al.^64^ and O’Sullivan et al.^45^. Removing these studies consistently weakened or eliminated the effects, likely reflecting variability in measurement devices rather than true physiological differences. Overall, the evidence for these three regions appears fragile and driven largely by a few influential studies.

All included studies involved participants of similar age with flexible flatfoot and used comparable single-session intervention protocols. Therefore, the differences observed in the forest plots are more likely to reflect true intervention effects rather than variability in sample characteristics. Under these conditions, exercise demonstrated the most consistent and clinically meaningful reduction in medial plantar pressure, supported by low heterogeneity across the contributing trials. In contrast, taping produced only modest and region-specific reductions, while insoles increased medial loading, indicating that exercise is the most effective intervention for reducing medial plantar pressure in individuals with flexible flatfoot.

However, different plantar-pressure devices with varying reliability were used across studies, making MDC values for flatfoot unclear and limiting interpretation of whether observed changes are clinically meaningful. This variability also complicates comparisons across studies and contributes to uncertainty regarding reported intervention effects.

Preliminary evidence indicates that conservative therapies—taping, insoles, footwear, and exercise—can modify plantar pressure, but without standardized measurement and longer follow-up, the durability and clinical relevance of these changes remain uncertain. Although several non-randomized studies suggest potential benefits, the overall strength of evidence is low to moderate. Despite moderate methodological quality in some studies (Downs & Black scores 9–14/15), their non-RCT designs remain prone to confounding and selection bias, limiting confidence in causal inference. Accordingly, these findings should be interpreted cautiously and verified through well-designed randomized trials.

### Clinical implications

First, use anti-pronation taping as an immediate diagnostic-therapeutic trial: if the patient experiences clear pain reduction or functional improvement within a few days of activity while taped, it indicates good potential responsiveness to mechanical interventions.

Simultaneously initiate and maintain an active strengthening program for the intrinsic foot muscles and tibialis posterior as the cornerstone of long-term management.

Prescribe insoles only after individual assessment using in-shoe pressure analysis or a very clear symptomatic response to temporary taping/padding; otherwise, there is a high risk of unwanted increased pressure in the medial midfoot region.

Implement all of the above within a comprehensive physiotherapy program that includes soft-tissue release techniques^76^, electrotherapy^77^, to maximize the likelihood of sustained effects.

Ultimately, until long-term, high-quality randomized controlled trials with standardized measurements and reported MCID/MDC become available, intervention selection must remain entirely patient-centered and based on continuous monitoring of individual response rather than assuming absolute superiority of any single method.

### Limitations and recommendations for future studies

Several limitations must be acknowledged in this systematic review. Primarily, the included studies lacked long-term follow-up data, with exercise interventions lasting a maximum of eight weeks and taping, footwear, and insole interventions examining only immediate effects. This limited duration constrains the evaluation of potential adaptations in connective tissue or muscular endurance over time, highlighting the need for future longitudinal studies to determine sustained efficacy. Methodological constraints also limit the conclusions: many studies had small sample sizes without power calculations, reducing external validity and weakening the strength of evidence. Additionally, while the Footscan system provides valid in-shoe pressure measurements, its technical limitations in capturing complete foot-surface contact may affect measurement precision.

Other limitations include insufficient investigation of dynamic tasks in exercise interventions, heterogeneity in foot posture assessment methods, potential publication bias (formal assessment was not feasible), and measurement bias related to variations in measurement tools across studies. These factors underscore the need for standardized protocols and careful interpretation in future research examining plantar pressure redistribution in pronated feet. Subgroup analyses were performed according to intervention type and study design, which were the only categories supported by the available data.

To address these limitations, future studies should conduct longitudinal randomized controlled trials lasting more than 12 weeks that simultaneously evaluate both biomechanical (e.g., plantar pressure redistribution) and symptomatic outcomes. We recommend further investigation into the synergistic effects of combined interventions, such as therapeutic exercise paired with orthoses or taping. Additionally, gait retraining^3^ and virtual reality-based exercises^78^ specifically tailored for flat feet are recommended. Employing standardized plantar pressure measurement platforms with minimal detectable change (MDC) reporting will enhance clinical interpretability. Finally, age- and sex-specific responses should be explored, as current evidence predominantly reflects middle-aged adults.

## Conclusion

Preliminary evidence suggests that conservative interventions, including exercise, taping, insoles, and footwear modifications, effectively promote a more physiological redistribution of plantar pressure in individuals with pronated feet. These approaches may help reduce excessive medial loading and support improved functional performance, highlighting their potential clinical value. However, current evidence remains limited by short-term follow-ups and variability in study designs. Future research should prioritize high-quality randomized controlled trials with longer intervention periods to establish optimal treatment protocols and assess sustained benefits.

## Supplementary Information

Below is the link to the electronic supplementary material.

Supplementary Material 1

Supplementary Material 2

Supplementary Material 3

## Data Availability

All data generated or analyzed during this study are included in this published article (and its Supplementary files).
