# Supplementary material for: Effects of conservative interventions on plantar pressure in individuals with flat foot: a systematic review and meta-analysis
Source: Sci Rep. 2026 Feb 19;16:9867. doi: 10.1038/s41598-026-40771-5 (PMC13018568; doi:10.1038/s41598-026-40771-5)
Supplement: Supplementary file 1 — Supplementary Material 1 [file 41598_2026_40771_MOESM1_ESM.docx]

| **Embase**  (pronation:ab,ti OR 'pronated foot':ab,ti OR 'pronated feet':ab,ti OR rearfoot:ab,ti OR 'flat foot':ab,ti OR 'flat feet':ab,ti OR 'pes planus':ab,ti OR 'arch collapse':ab,ti OR planovalgus:ab,ti OR 'flat arched feet':ab,ti OR 'pes planovalgus':ab,ti OR eversion:ab,ti OR inversion:ab,ti OR 'low arched feet':ab,ti OR 'excessive calcaneal eversion':ab,ti OR 'medial longitudinal arch':ab,ti) AND (shoes:ab,ti OR taping:ab,ti OR insole:ab,ti OR excercise:ab,ti OR training:ab,ti OR intervention:ab,ti OR therapeutic:ab,ti OR treatment:ab,ti OR modification:ab,ti OR retraining:ab,ti OR 'physical therapy':ab,ti OR 'orthosis':ab,ti) AND (pressure:ab,ti) |
| --- |
| **Scopus**  "pronation" OR "pronated foot" OR "pronated feet" OR "rear foot" OR "flat foot" OR "flat feet" OR "pes planus" OR "arch collapse" OR "planovalgus" OR "flat arched feet" OR "pes planovalgus" OR "eversion" OR "inversion" OR "low arched feet" OR "excessive calcaneal eversion" OR "medial longitudinal arch"  AND  "shoes" OR "taping" OR "insole" OR "exercise" OR "training" OR "intervention" OR "therapeutic exercise" OR "physical therapy" OR "modification" OR "treatment" OR "retraining" OR "orthosis"  AND  “pressure” |
| **PubMed**  "pronation”[Title/Abstract] [Title/Abstract]OR "pronated foot”[Title/Abstract] OR "pronated feet”[Title/Abstract] OR "rear foot”[Title/Abstract] OR "flat foot”[Title/Abstract] OR "flatfoot”[Title/Abstract] OR "flat feet”[Title/Abstract] OR "flatfeet”[Title/Abstract] OR "pes planus”[Title/Abstract] OR "arch collapse”[Title/Abstract] OR "planovalgus”[Title/Abstract] OR "flat arched feet”[Title/Abstract] OR "pes planovalgus”[Title/Abstract] OR "eversion”[Title/Abstract] OR "inversion”[Title/Abstract] OR "low arched feet”[Title/Abstract] OR "excessive calcaneal eversion”[Title/Abstract] OR "medial longitudinal arch"  AND  "Shoes”[Title/Abstract] OR "footwear”[Title/Abstract] OR "insole”[Title/Abstract] OR "taping”[Title/Abstract] OR "tape”[Title/Abstract] OR "exercise”[Title/Abstract] OR "training”[Title/Abstract] OR "intervention”[Title/Abstract] OR "therapeutic exercise”[Title/Abstract] OR "physical therapy”[Title/Abstract] OR "modification”[Title/Abstract] OR "treatment”[Title/Abstract] OR "retraining”[Title/Abstract] OR "conservative”[Title/Abstract] OR "orthosis”[Title/Abstract]  AND  "Pressure"[Title/Abstract] |
| **Web of Science**  Title="pronation" OR "pronated foot" OR "pronated feet" OR "rear foot" OR "flat foot" OR "flatfoot" OR "flat feet" OR "flatfeet" OR "pes planus" OR "arch collapse" OR "planovalgus" OR "flat arched feet" OR "pes planovalgus" OR "eversion" OR "inversion" OR "low arched feet" OR "excessive calcaneal eversion" OR "medial longitudinal arch"  AND  Topic="Shoes" OR "footwear" OR "insole" OR "taping" OR "tape" OR "exercise" OR "training" OR "intervention" OR "therapeutic exercise" OR "physical therapy" OR "modification" OR "treatment" OR "retraining" OR "conservative" OR "orthosis"  AND  Topic="Pressure" |

**Table S1. Search strategy for 4 databases**
