# Supplementary figures and images for: Effects of conservative interventions on plantar pressure in individuals with flat foot: a systematic review and meta-analysis
Source: Sci Rep. 2026 Feb 19;16:9867. doi: 10.1038/s41598-026-40771-5 (PMC13018568; doi:10.1038/s41598-026-40771-5)

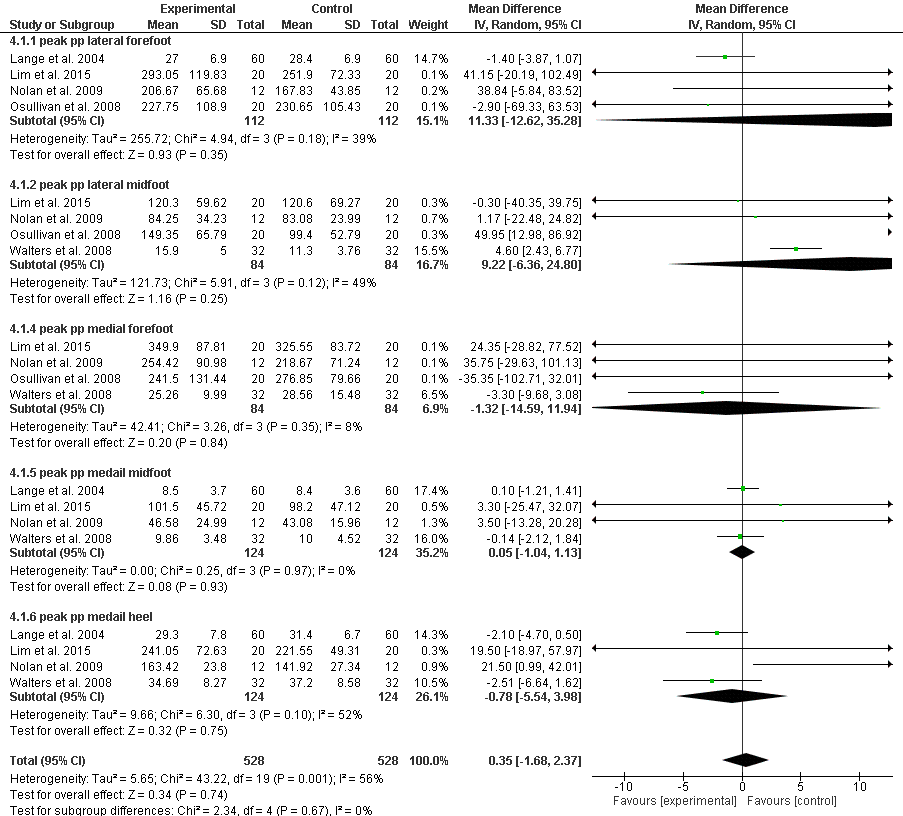


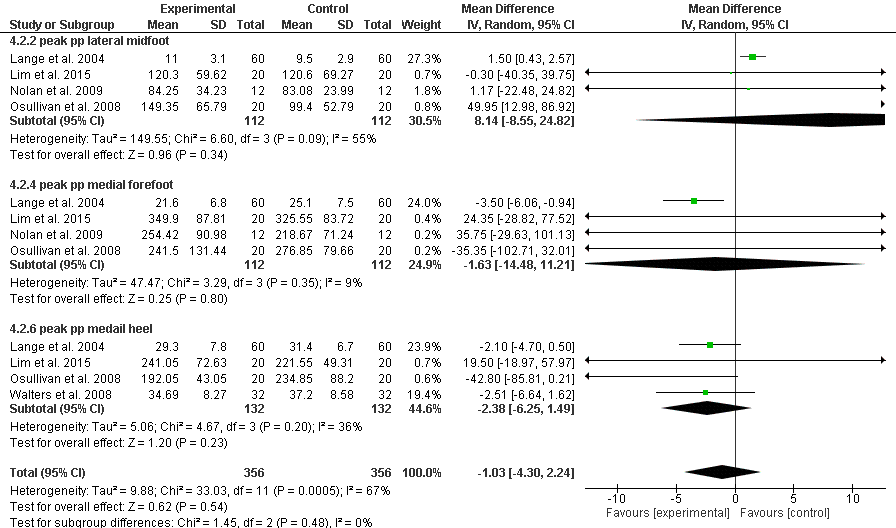


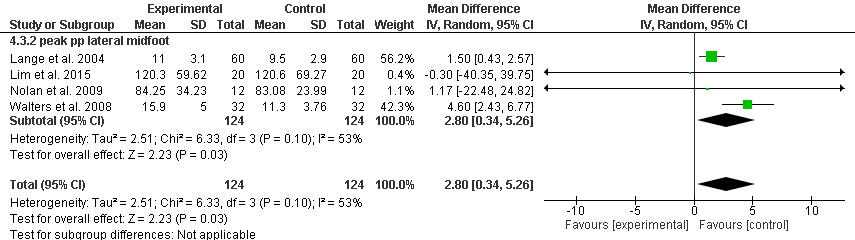


**Fig S1. Sensitivity analysis results after removing studies one by one for Taping**

Supplement: Supplementary file 2 — Supplementary Material 2 [file 41598_2026_40771_MOESM2_ESM.docx]
